# A Valuable Invention

**Published:** 1881-09

**Authors:** 


### A VALUABLE INVENTION.

Should M. Gentilli, of Leipsic, succeed in persuading members of Parliament, popular preachers and other public speakers in different parts of the world to deliver themselves of their sentiments and opinions for the future through an apparatus recently invented and patented by him, stenography will soon be an obsolete science, and the short-hand writer will have reason to lament, like Othello, that his "occupation's gone." Something between a mouth-organ and a jew's-harp is to be lightly held between the teeth by the orator while speaking.

This mechanism is connected by electric wires with a metal roller, upon the exterior surface of which, as it revolves, the utterances of the person disclaiming through the apparatus appear in luminous characters as fast as they are spoken. Possibly the spectacle of a legislator performing his speech, as it were, upon an instrument beginning like a hautboy and ending like a printing-press, might appear somewhat indecorous at first, but we might soon become accustomed to it, and the blessings of Gentilli's apparatus would more than compensate those using it for any trifling ridicule they might have been subjected to while it was still an absolute novelty.—*London Telegraph.*

